# You can’t burn the house down because of one bedbug: a qualitative study of changing gender norms in the prevention of violence against women and girls in an urban informal settlement in India

**DOI:** 10.12688/wellcomeopenres.11805.2

**Published:** 2017-11-07

**Authors:** Nayreen Daruwalla, Ketaki Hate, Preethi Pinto, Gauri Ambavkar, Bhaskar Kakad, David Osrin

**Affiliations:** 1 SNEHA (Society for Nutrition, Education and Health Action), Urban Health Centre, 60 Feet Road, Dharavi, Mumbai, Maharashtra, 400017, India; 2 University College London Institute for Global Health, 30 Guilford Street, London, WC1N 1EH, UK

**Keywords:** India, Mumbai, poverty areas, violence, social norms, gender role

## Abstract

**Background**: The contribution of structural inequalities and societal legitimisation to violence against women, which 30% of women in India survive each year, is widely accepted. There is a consensus that interventions should aim to change gender norms, particularly through community mobilisation. How this should be done is less clear.

**Methods**: We did a qualitative study in a large informal settlement in Mumbai, an environment that characterises 41% of households. After reviewing the anonymised records of consultations with 1653 survivors of violence, we conducted 5 focus group discussions and 13 individual interviews with 71 women and men representing a range of age groups and communities. We based the interviews on fictitious biographical vignettes to elicit responses and develop an understanding of social norms. We wondered whether, in trying to change norms, we might exploit the disjunction between descriptive norms (beliefs about what others actually do) and injunctive norms (beliefs about what others think one ought to do), focusing program activities on evidence that descriptive norms are changing.

**Results**: We found that descriptive and injunctive norms were relatively similar with regard to femininity, masculinity, the need for marriage and childbearing, resistance to separation and divorce, and disapproval of friendships between women and men. Some constraints on women’s dress and mobility were relaxing, but there were more substantial differences between descriptive and injunctive norms around women’s education, control of income and finances, and premarital sexual relationships.

**Conclusions**: Programmatically, we hope to exploit these areas of mismatch in the context of injunctive norms generally inimical to violence against women. We propose that an under-appreciated strategy is expansion of the reference group: induction of relatively isolated women and men into broader social groups whose descriptive and injunctive norms do not tolerate violence

## Introduction

Across the world, women continue to suffer physical, emotional, sexual, and economic violence ^1^. Preventing such violence has been a World Health Organization priority since 2013 ^2^, and is a target for the fifth Sustainable Development Goal. The contribution to endemic violence of structural inequalities and implicit and explicit legitimisation is now widely accepted ^3^. Contemporary prevention programs attempt to address determinants such as patriarchal arrangements, hegemonic masculinity, and inequitable gender roles. A common way to understand the determinants of violence against women and girls is to frame them in a socio-ecological model that locates individual personal histories within families, located in turn within communities, and in turn within societies ^4, 5^. There is broad agreement that interventions should operate at multiple levels, from individual to societal. Interventional discourse has also moved along this path, from a concentration on the needs of survivors of violence to an acknowledgment that intervention should aim to “transform the relations, norms, and systems that sustain gender inequality and violence” ^6^. Of particular interest are gender norms that privilege controlling and aggressive behaviour in the prevailing template for masculinity ^6, 7^. Efforts to change them are usually termed ‘gender transformative’ ^8^.

A social norm is a belief in the expectations of others in a social group ^8– 11^. It is maintained by the influence of a reference group of people important to an individual’s decision-making ^8, 10^. One conceptual norm - a descriptive norm - describes beliefs about what others actually do (roughly equivalent to an empirical expectation or a collective behavioural norm) ^12– 14^. A second – injunctive - norm describes beliefs about what others think one ought to do (equivalent to a normative expectation or a collective attitudinal norm) ^12, 14^. Cialdini, Kallgren, and Reno contrast these two ideas as what is commonly done and what is commonly approved, or ‘is’ and ‘ought’ ^12^. An important aspect of a social norm is that it describes what people perceive as the beliefs of the reference group around them, regardless of whether their perception differs from reality. For example, many people in a group may disagree with a norm, but think that others support it. This failure to recognise private disagreement with a perceived norm has been called pluralistic ignorance ^9, 15, 16^, and might at least partly explain why behaviours are sustained when people privately disapprove of them ^9^.

Why we do what we do is complicated and norms are only part of the story. For example, a behaviour might be sustained or prevented by social structures such as laws and institutions, material contributors such as wealth (or lack of it), or the availability of services. It might equally be driven by personal beliefs, self-confidence, and aspirations ^17^. From an economic perspective, norms are supported by coordination, social pressure, signalling, and anchoring. Coordination allows individuals to express themselves in shared languages – literally and metaphorically – and benefits both them and the collective. Social pressure encourages individuals not to act purely in their own interest, but that of the collective. Signalling and symbolism allow individuals to identify with (or, equally, indicate their lack of identification with) social groups. Anchoring effectively sets benchmarks for behaviour within a smaller range than what is possible, an example being the ages at which women and men marry ^18^. Levy Paluck and colleagues suggest that norms have a stronger influence on an individual’s behaviour if they have a clear central tendency (what people do is similar to what they believe others think they ought to do: descriptive and injunctive norms are similar), show little dispersion (such as variation from place to place), and are ascribed to a reference group important to the individual. The greater its importance in her everyday life, the stronger the adherence to a social norm is likely to be ^9^.

The resistance of social norms to change varies. They may be sensitive to changes in social networks and to the influence of individuals who emerge as role models or deviants ^17^, and their supporting matrix may be complex. For example, violence against women is unlikely to be sustained by a single norm and often occurs at the intersection of gender norms that are permissive rather than supportive ^17^. A gender norm is a kind of social norm that describes shared social expectations of behaviour specific to gender ^19^. It tends to emerge from gender ideology and attitudes; for example, valuing sons over daughters, idealised conceptions of femininity, and traits that signal masculinity. Glibly, we might think of these as archetypes of the *good woman* and the *real man*: constructs that may hinder change rather than actively support violence ^20^, but contribute to imbalances of power. These have themselves led to socially constructed gender roles. Most of our discussion is about gender norms that legitimise imbalances of power and, by extension, inequalities in access to resources ^19, 20^.

Marcus and Harper suggest that gender norms are more likely to change when no parties have strong economic interests at stake, no one’s power is directly threatened by change, one key factor underpins a norm, there are no religious injunctions to continue a certain practice, role models and opinion leaders promote changed norms, a changing institutional or political context provides opportunities for changed practices, and norm change communications are paired with opportunities for action ^21^. A norm that constrains some people, such as denying education to girls, benefits others and it is important to find common ground inasmuch as the perceived net benefit of norm change is positive ^20^.

We developed a study to understand them in the context of an urban informal settlement in India, asking three questions: whose opinion matters (the reference group), is the behaviour believed to be typical of the reference group (descriptive norm), and is it believed to be appropriate (injunctive norm)? ^8, 10^

## Methods

### Setting

India illustrates the nexus of gender inequality, sociocultural legitimisation, and violence. Intimate partner violence is endemic and sexual violence in public spaces is reported regularly in the media ^22^. A recent systematic review suggested that 22% of women had survived physical abuse in the past year, 22% psychological abuse, 7% sexual abuse, and 30% multiple forms of domestic violence ^23^. Unequal norms appear to be associated with intimate partner violence ^24, 25^. The latest National Family Health Survey suggests that 21% of ever-married women in Maharashtra state have experienced spousal violence ^26^. SNEHA (Society for Nutrition, Education and Health Action) is a secular non-profit organisation in Mumbai. Our Program on Prevention of Violence against Women and Children aims to develop strategies for primary prevention, ensure survivors’ access to protection and justice, empower women to claim their rights, mobilise communities around ‘zero tolerance for violence’, and respond to the needs and rights of excluded and neglected groups. Our community mobilisation work is part of a broader package that includes support for individual survivors and collaborative efforts to improve the response to violence by police and healthcare workers.

Two-thirds of cities and towns in India include informal settlements (slums) ^27^, characterized by overcrowding, insubstantial housing, insufficient water and sanitation, lack of tenure, and hazardous locations ^28, 29^. There will be over 100 million people in such settlements by 2017 ^30^, and they currently include 41% of Mumbai’s households ^27^. Our program has been working in Dharavi, one of Asia’s largest informal settlements, since 2000. The population includes a range of cultural and religious groups, whose diversity is illustrated by the availability of schooling in six languages. Some of the 82 geographical clusters are homogeneous, retaining cultural group identity. Others are heterogeneous, but all embody the idea of villages within the city ^31^. This, and factors like poverty, poor housing and environment, and governmental neglect, make Dharavi a backdrop for the operation of identity politics which are predominantly communal and patriarchal.

### Study procedures

Before data collection, we convened a group of fieldworkers and counsellors to develop a provisional set of examples of social norms, using anonymised existing records from 1653 clients registered at our crisis and counselling centre in 2012–2015. Counsellors then selected cases purposefully to reflect a range of presenting problems (for example, natal family violence, intimate partner or domestic violence). This preliminary exercise reached saturation after detailed discussion of 25 cases ( Table 1). While most of the examples were social norms, a few – “boys will be boys,” “women should not have sex before marriage,” and “it is a good woman’s duty to make her husband’s family happy” - had the character of more general moral norms. Having identified problems presented by age, religion, caste, employment, and wealth, we developed a fictional vignette for discussion, covering premarital life, getting married, and marital life.

**Table 1.** Provisional normative statements to inform focus group discussions and interviews, developed from thematic analysis of anonymised client records, 2012–2015.

Violence	It is sometimes acceptable to hit a woman. Men do not think physical and sexual violence against their wives is an issue. Violence is often blamed on external factors like the woman’s parents. It is traditional for boys to pester girls. Boys will be boys.
Youth	A family, especially male relatives, are entitled to control a girl’s life. It is acceptable to pressure a woman to get married. A pregnant daughter-in-law is the responsibility of her natal family. Parents can assault their daughter if she transgresses acceptable behaviour. Parental control over an adult daughter is acceptable. Women should not have sex before marriage.
Arranged marriage	Arranged marriages are acceptable. A marriage may be arranged without disclosing to the woman’s family potential weaknesses in her husband. Dowry is appropriate, even if the woman is more educated and well-to-do than the man. Aggravated violence and threats to a woman’s life are acceptable if she brings insufficient dowry.
Love marriage	A woman should hide abuse from her natal family if they don’t approve of her partner. The natal family does not have to support a woman if she has had a love marriage and is then subjected to violence.
The good wife	It is a good woman’s duty to make her husband’s family happy. It is a good woman’s duty to make her marriage work, even by returning to an abusive husband. A woman should not react to violence. A woman should take back her abusive husband when he promises to behave in future. Marital rape is acceptable. A woman owes a man sexual pleasure. A woman’s physical mobility may be restricted. A woman’s contact with her natal family may be restricted. A daughter-in-law should listen to her in-laws. Male family members may feel entitled to have a sexual relationship with a woman who enters the family, and this may be acceptable to other affinal women. The natal family should support the couple when the husband is incapable of being the provider. A family may legitimately control the behaviour of a daughter-in-law and her husband. A husband should support his family over his wife.
Children	Abuse is justifiable if a woman does not conceive. Abuse is justified if a woman has a girl or an unhealthy child. A man does not have child-rearing responsibilities.
	A child may be used as leverage over a woman after she has left an abusive situation. It is acceptable for in-laws to retain custody of a male (grand)child after a woman separates from her husband.
Police	Police see domestic violence as a private matter and do not necessarily take it seriously. Police are reticent to take action that is challenging. It is acceptable for the police to call a woman’s parents, irrespective of her views.
Beyond the family	Community leaders have a say in issues such as marriage.

Responses to survey questions on gender norms – such as those used in Demographic and Health Surveys – may differ from responses to more contextualised questions based on illustrative vignettes ^32^. Over three months in 2016, we held a series of focus group discussions and individual interviews. A purposive sample of female and male participants represented two age groups (18–30 and 30–55 years) and four localities. We intentionally included more women than men because our preventive program works primarily with women as drivers of norm change. A team of ten went door-to-door to recruit participants from pre-selected regional, religious, or cultural communities, mobilising women by age group as participants for focus group discussions. Simultaneously, counsellors referred clients by age group and community. Three postgraduate female researchers, already working with the program and with experience of qualitative research, conducted focus group discussions and interviews with women. Men were interviewed by a graduate male community worker. Because we wanted to minimise social desirability bias as a result of previous exposure to program activities, we invited people unfamiliar with our work to participate. Participants and researchers generally met for the first time at discussions and interviews. The researchers talked about their professional backgrounds, experience, and the reasons for the study. They described violence as potentially affecting all women, including themselves, and urged participants to speak candidly so that their opinions could be used to design interventions that would help others. They assured participants that they could contact a supervisor if they had concerns.

Focus group discussions were held in community spaces familiar to participants: a program community centre, the homes of community volunteers or participants, and a temple. Interviews with women were conducted at a municipal Urban Health Centre. Women often brought children and grandchildren to discussions and the researchers provided drawing materials to occupy them. Women who did not fit the focus group age bracket were often present.

The vignette used in focus group discussions followed a hypothetical biography as a means of eliciting opinions ^8^, and was piloted with a mixed group of male and female staff *.* It traced a woman’s life from later schooldays until about ten years into her marriage. Example scenarios included having a boyfriend whom her parents did not know about, her parents checking her mobile phone, not wanting to have children soon after her wedding (when her in-laws wanted her to), wanting in-laws to take care of her first child so that she could complete her education, her husband realising she had male friends at college, her desire to give some of her salary to her parents, being delayed at work and not preparing food for her husband and children, her husband suspecting that she was communicating with another man by smartphone, and discovering that her husband was having an affair.

Interviews followed a semi-structured topic guide in which participants were asked to give a biographical account of their lives. Discussions and interviews were audio-recorded and researchers took notes to triangulate transcription and translation. Transcripts were translated from Hindi and Marathi to English by KH and an independent translator, with review for accuracy by PP. The average duration of focus group discussions was 53 minutes, and of interviews 44 minutes. Data saturation was discussed when designing the study and during the course of data collection. Participants were given the option of reviewing the recordings or transcripts of their interviews, but none requested to do so.

### Ethical statement

The study was approved by the ‘Ethicos’ Independent Ethics Committee, Mumbai, on 3rd December 2015. We reviewed our existing case records to identify norms for consideration. Signed informed consent is taken from all clients when they first access our services. Counselors inform them of their right to access their records in building evidence for their legal cases. They are told that their anonymised information may be used in research conducted by the organisation, with the aim of improving services. Clients are assured of confidentiality, particularly that information will not be shared with the perpetrator’s family, community members, or the media.

Participant information sheets and written consent forms (in English and Hindi, read aloud when appropriate) were given to participants before interviews and focus group discussions, and all participants provided signed consent. Although the interviews and focus groups did not consider participants’ own experiences, interviewers were familiar with existing organisational response and referral protocols and followed WHO recommendations for research on violence against women ^33^. Participants were advised that they should consider as confidential any information shared by other participants in focus groups that might have referred to local individuals.

### Analysis

We developed a framework analysis, beginning with a provisional set of norms, attitudes and beliefs identified from the literature, case review, and practitioner workshop. We used framework analysis because we came to the study with a provisional classification of norms, and had an agreed sample and timeline ^34, 35^. Transcripts were analysed in NVivo 10 ( www.qsrinternational.com). Because focus groups and interviews followed a semi-structured sequence, we began with a list of general coding categories, which we expanded and sub-categorised into a coding tree in a series of team discussions ^36– 39^. These categories described types of gender norm, response to transgression, and classification as descriptive or injunctive. We revisited the analysis repeatedly over six months, reviewing individual transcripts and hierarchies of categorical codes in an effort to achieve a higher level of thematic description. We settled on the comparison of descriptive and inductive norms early, but the idea of the importance of reference groups emerged much later.

## Results

### Areas in which injunctive and descriptive norms coincided: femininity, masculinity, marriage, childbearing, separation, and friendship across sexes

Table 2 summarises discussions with 56 women and 15 men between 30 ^th^ September and 23 ^rd^ December, 2015 (10 interviews and 8 focus group discussions), which supported views of women’s and men’s roles familiar from our work and the literature. Older men and women favoured arranged marriages, a belief in which descriptive and injunctive norms coincided. They said that a woman became the responsibility of her in-laws after marriage and that her mobility, even to her natal home, needed to be monitored. While intimate partner and domestic violence were disapproved of, most participants said that some violence was acceptable if a woman neglected the house and children, did not cook properly, was unfaithful, or disrespected her in-laws. Shouting and slapping were acceptable, but anything more was not. Older men said that, as household heads, their masculinity depended on their roles within and outside the home.

**Table 2.** Focus groups and interview participants.

Community affiliation	Focus group participants	Individual interview participants	*Total*
1 Hindu	26 women	4 women	*30 women*
	10 men	1 man	*11 men*
2 Muslim	6 women	4 women	*10 women*
	3 men	1 man	*4 men*
3 Tribal	7 women	1 woman	*8 women*
4 Christian	6 women		*6 women*
5 Buddhist		2 women	*2 women*
**Total**	**58**	**13**	***71***

“Men have to earn, take care of the family, keep the people around organized and united. A lazy man won’t be accepted by society and people will taunt him.”

                                                                                      Interview, older Hindu man (OM-I-89)

Masculine behaviour was upheld by social sanctions. Younger men and women agreed that there was nothing wrong with a man cooking or helping his wife with domestic chores, but men refrained from doing so because they thought they would be gossiped about and taunted.

*R2 (Respondent 2): I will laugh at him.*
*I (Interviewer): What will make you laugh at him?*
*R2: Because even though he has a wife he is cooking food.*
*R5: Yes, people make fun and laugh because a woman should prepare food and here he is, a man who’s cooking food.*

                                                                                      FGD (Focus group discussion), older men (OM-F-78)

Women agreed that men preferred not to help because they were conscious of their peers and families impugning their masculinity. Participants often defined masculine behaviour against the construct of the good woman, for whom the level of sanction was more punitive: participants in focus group discussions said that it was acceptable to beat a woman if she did not conform to the archetype of a good daughter, wife, mother, or daughter-in-law. Injunctive and descriptive norms coincided in the belief that women should get married and have children, irrespective of their education and income and whether or not they married willingly. In one community, adolescent marriage was still common. Mothers were not keen to relax this expectation, even though it was somewhat at odds with their belief that girls should be educated:

*I: How old is your daughter?*
*R: Ten.*
*I: Do you think you’d like to her to get married?*
*R: She is studying now. When she grows older and finishes her schooling, I’ll get her married. She will not get married early like us. We were married when I was thirteen. It was way too early, but now there are rules to let her study and become independent. When she agrees we will get her married; not before that.*
*I: At what age do you want to get her married?*
*R: Maybe at fifteen or twenty, whenever she wants to.*

                                                                                      Interview, older Hindu woman (YW-I-43)

Women said that they based the decision to marry on what their families thought. If parents found out that their daughter had a boyfriend, she would often face sanctions. Her education would be terminated and her mobility would be restricted. Sanctions against women for not conforming to marriage norms were stronger than they were for men.

*I: What happens if a girl decides not to marry?*
*R: They call her names and say things like, she isn’t married or she hasn’t found a guy yet.*
*I: What if a man isn’t married?*
*R: Nothing. They only say things to a woman. For men it’s easy because they think that they have several options to pick any woman.*

                                                                                      Interview, younger Hindu woman (YW-I-12)

Women said that they, or people they knew, often stayed in abusive marriages for fear of the implications of separation. They had seen or heard of divorced women being gossiped about, taunted, and abused. This was a strong reason for staying, apart from factors such as financial dependence on the perpetrator, lack of confidence, and worry about the children. A woman who separated from her husband was expected not to talk to men unknown to the family, not to entertain proposals from men, and to cater to her children’s needs above all else. Both injunctive and descriptive norms were for her not to divorce. If she did, the injunctive norm was for her to return to her natal family, who might or might not support her decision. The commonest sanction for transgression was reputational damage.

*I: What will happen if she decides to separate from him?*
*R4: She will not be able to live happily in this society because in such cases no one supports you, neither your parents nor your in-laws. If nobody supports her what will the girl do? Mostly those girls commit suicide.*
*I: There are several women who live alone by themselves.*
*R3: People taunt. They say that she doesn’t have a husband and does immoral things. Even if she earns an honest living, they assume that she is doing some dirty work.*
*I: What do you mean by dirty work?*
*R4: Like talking to people outside and having affairs with men.*

                                                                                      FGD, younger women (YW-F-52)

Women almost all said that they did not have male friends when they were young for fear of retribution from male relatives. They said that men were quite comfortable with beating female relatives for transgression.

*R: My brothers didn’t allow me to make friends with other girls because they thought I might do the same things like talking to and going around with boys. I wasn’t crazy enough to do anything like that because I knew how my brothers are.*
*I: So you wouldn’t go with them because you were scared of your brothers?*
*R: Yes.*
*I: What would happen if you were friends with boys?*
*R: They would’ve beaten me if I were to even speak of being friends with boys.*

                                                                                      Interview, younger Hindu woman (YW-I-11)

During a discussion on premarital sex, older women said that girls faced violence because their actions were wrong and boys were often beaten because they had tainted the girl’s family honour.

*R6: When daughters do bad things, our name gets soiled. The girl is humiliated because she has been with some boy. People in the community say, “We saw her roaming with this boy. Maybe she has an affair with him.” Even if he’s just a best friend and not a boyfriend, it all depends on the eyes of the beholder. People don’t assume good things about women, they only assume bad things like she has a boyfriend.*

                                                                                      FGD, older women (OW-F-51)

Some women interviewees said that they were aware of, or were suspicious of, their husbands having sex outside marriage and seemed to accept it. The same women, however, had approached our organisation because their husbands had physically abused them when they thought they were talking to other men. Only one woman said that she had had an extramarital relationship. The acceptance with which wives treated their husbands’ behaviour did not extend to her. Control of sexual activity included sanctions against women using mobile phones. Older women said that only women who were “up to no good” needed “advanced phones” because they used them to exchange messages and photos with other men. Interviewees who had been in abusive marriages said that they would be physically and verbally abused for using a mobile phone.

*My husband didn’t like me using a mobile phone, so I didn’t use one for a year. However, he used to grudgingly agree to let me use his mobile phone if I wanted to call. Once I was done using his phone, he’d ask, “What have you told your mother? How many of your boyfriends have you sent messages through her?” And then he used to beat me.*

                                                                                      Interview, younger Hindu woman (YW-I-12)

### Areas in which injunctive and descriptive norms differed slightly: dress, mobility, and visibility

Older men said that women from their area who dressed inappropriately invited sexually coloured remarks and that good women dressed appropriately. In some cases, however, this attitude was relaxed toward women perceived as higher-class.

*In our society, good girls shouldn’t wear sexy clothes. If you’re fully covered then it’s better for your safety. In rich societies you will find girls wearing short skirts and roaming around with rich boys, but they aren’t wrong. The poorer the girl, and if she has a boyfriend, then…*

                                                                                      FGD, older men (OM-F-78)

Discussions of the veil came up with both Hindu and Muslim interviewees. Women said that the norm dictating that they wear it had relaxed, while men were more in favour of maintaining it. Men considered themselves protectors of women and community honour, and saw the veil as a means of protecting ‘their’ women from other men. Women’s safety was linked with ‘appropriate’ clothing, of which the veil was an important component.

*If women remove the veil, it won't be good for them. The veil is an adornment for a woman. If they don't wear it then they will get the evil eye ... If they don't wear the veil outside then boys and men will talk about them and word will spread in society that they are like those other women who don’t wear the veil.*

                                                                                      Interview, older Hindu man (OM-I-89)

When women said that the expectation of wearing the veil had relaxed, they meant that they were not required to wear it at home, but were expected to wear it in public. Many did so, fearing negative sanctions like gossip and taunts from neighbours and family.

*R: Before marriage I used to wear a burkha, but only when I went to work. In our culture, when a girl starts menstruating, she has to wear a burkha.*
*I: Now where do you wear it?*
*R: I wear it when I am going outside the house. Sometimes I don’t wear it when I am just going to the shop. Anyway, I don’t even like to wear it.*
*I: Then why do you wear it?*
*R: Because neighbours and family will talk about it.*

                                                                                      Interview, younger Muslim woman (YW-I-92)

The main sanction appeared to be name-calling and reputational damage, the results of which could be tragic:

*I: What happens to women who don’t conform?*
*R: People taunt. Women from any religion sleep with men for money, some work in bars, some even have more than one boyfriend. So people try and talk to them. If they don’t listen, they are taunted and verbally abused.*
*I: Do these taunts make her change her ways and make her wear purdah?*
*R: Some women get tired and decide to change their ways. A woman thinks that she can’t burn the whole house because of one bedbug. She has to live her life as well; she has people to take care of. Instead of listening to people’s taunts, she may as well do the purdah. Some people commit suicide because they’re mentally stressed as they are not allowed to live according to their wish. It’s good if you do purdah. Which man would want another man to cast a glance at his woman? No one would want it. No man from any religion would be able to tolerate another man being able to see a woman’s figure.*

                                                                                      Interview, younger Muslim man (YM-I-14)

### Areas in which injunctive and descriptive norms differed markedly: education, earnings, premarital relationships, and premarital sex

As with employment, there was a clear impression of change in norms around female education. Participants were unanimous in their belief that the practice of not educating girls had to change. They also supported the idea of continuing education after marriage and childbirth.

*The in-laws need to understand and treat their daughter-in-law like their own daughter. If she wishes to study then they should allow her because eventually children will be born. It’s not like she is running out of time due to her age...*

                                                                                      FGD, younger women (YW-F-20)

This commitment to women’s self-determination wavered when the demands of education compromised their ability to fulfil gendered family responsibilities.

Men reported relatively progressive attitudes to gender roles in decision-making. Although older men stressed that husbands should be providers, they said that wives should have control over their own earnings. A woman could decide what to do with income: either contribute to household expenditure or save for an emergency. Emergencies were themselves gendered, relating to the woman’s reproductive role, examples being children’s illnesses or demands for school fees. Although participants mentioned that women’s expenditure had traditionally been controlled by their families, the idea of a woman earning her own money was nothing special. There were, however, limits: interviewees found the idea of a woman spending her entire income on her own needs selfish. The ‘traditional’ norm was for the natal family to have no call on a woman’s income once she entered her in-laws’ home, but younger men and women said that she had the right to choose whether she wanted to give part of it to her own parents.

*Her in-laws should not have an eye on her salary because it is her money and she has earned it. It is a good thing that she is giving it to her in-laws and also to her parents because they have educated her since her childhood. So it is correct if she gives some to her mother and keeps some for herself. If someone toils hard every month to earn money and if that person doesn’t get to spend even a rupee, then how will that person feel?*

                                                                                      FGD, younger men (YM-F-97)

One interviewee, who had been separated from her husband for more than a decade, was planning to get back together with him, but said that she would not continue to work.

*If I start working he will become lazy because he won’t realise his responsibilities. Nobody was there to support me, so I had to earn for my child’s education. Now I want him to take up all the responsibilities. Whatever money he sends me, I’ll manage to feed my child and look after him in that amount.*

                                                            Interview, older Muslim woman (OW-I-46)

Rather than being discouraged, women were nowadays encouraged to take financial responsibility, although the potential sources of income were constrained.

*R: My husband earns and I run the household. He doesn’t even go to the market. He earns 7000 Rupees, he gives me 6000 and keeps 1000 for his personal expenses. He asks me to do whatever I please with the money.*

                                                                                      FGD, younger Muslim woman YW-I-92

The idea that young people should take seriously the advice of their elders on relationships and sex was more of an injunctive than a descriptive norm, and it happened rarely. Although not entirely comfortable with premarital sex, older men and women seemed to be resigned to the fact that young girls and boys were sexually active and were making sexual decisions irrespective of their marital status.

*R2: All of them have sex. They don’t wait till eighteen, they start at thirteen.*
*R3: Yes, they start at thirteen.*
*R2: No one waits till eighteen. The rate at which they go, they can have two children by the age of eighteen.*

                                                                                      FGD, older women (OW-F-51)

Women’s sexuality was bound up with family status. The primary responsibility for premarital sex fell on girls and women because of fear that an unwanted pregnancy could dishonour the family.

### The importance of reference groups

Across age groups, women and men described their reference groups as ‘society’ or ‘community’. What these words meant was less clear, as we have found in other research ^40^. Women interviewees often talked about society as an abstract body that dictated their decisions, mobility, and responsibilities, but struggled to define it further.

*R: ... we do this because of society.*
*I: What is society?*
*R: What do you mean?*
*I: You tell me.*
*R: I don’t know.*

                                                                                      Interview, older Muslim woman (OW-I-46)

Although these words call to mind large collectives, their interpretations were actually quite limited. For women, the community was the family. Men seemed to have a broader conception of community and society, possibly because of their mobility and social interactions. Hindu and Muslim women said that they respected the authority of elders from the Panchayat and the Jammat, respectively, and men talked about performing gender roles according to the norms dictated by religious bodies. Older men said that their behaviour was often shaped by the opinions of those in power within these local organisations. There were gendered differences in the behaviour of such bodies. Younger men were more cynical about traditional authorities and their influence on gender roles and ideologies.

*Society laughs at other people's misfortunes and is unhappy at your success. Your behaviour should be in accordance to religious leaders and your parents.*

                                                                                      FGD, younger men (YM-I-14)

Women’s behaviour and decisions were dictated more by their families - natal families before marriage and affinal afterwards – and by the notion of reputation among neighbours.

*I: We often use the word community or society when we…*
*R: When we often don’t say or do particular things because of the fear of others.*
*I: Who are these others?*
*R: These are different types of people. They are our neighbours. My in-laws don’t like me socialising with people from the locality. That’s why I rarely talk to my neighbours. And if the neighbours come to know about our fights, they may talk about us and discuss our situation behind our backs.*

                                                                                      Interview, younger Hindu woman (YW-I-11)

Women often took pride in the fact that they did not interact with people outside their families, a trait that made them good women in family eyes. This extended to abandoning friends, particularly in the case of male friends after marriage, which further reduced their networks of interaction and narrowed their reference group.

*I: Who is part of this society?*
*R: Just my mother and father and no one else. I don’t trust anyone, nor do I talk to anyone. I just mind my work, my kids and that’s it. People keep talking about something happening somewhere.*
*I: Are these people a part of society?*
*R: Yes.*
*I: I’m not referring to your family here… for you what comprises a society?*
*R: I don’t know what to say as I told you, I stay indoors mostly and I don’t go out.*

                                                                                      Interview, younger Hindu woman (YW-I-26)

We take two ideas forward from these illustrations. First, reference groups were tight and generally involved strong social ties (families, local cultural and religious organisations) ^41^, which we might think of in terms of bonding social capital ^42^. For women, they were extremely tight and often confined to close family. Men described wider networks, albeit still fairly local. These reference groups were conduits for the opinions of a wider reference group, locally in the form of neighbours and distantly in something called society. Our inference is that these closely bonded reference groups were likely to transmit – and transmute - injunctive norms from a dimly perceived wider world.

## Discussion

**Figure 1.**
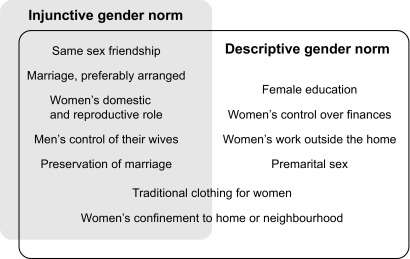
Degree of overlap between injunctive and descriptive gender norms.

Focus group discussions and interviews involving 71 women and men in an urban informal settlement in Mumbai suggested that injunctive and descriptive norms were relatively similar with regard to femininity, masculinity, the need for marriage and childbearing, resistance to separation and divorce, and disapproval of friendships between women and men. Some constraints on women’s dress and mobility were relaxing, but there were more substantial differences between injunctive and descriptive norms around women’s education, control of income and finances, and premarital sexual relationships ( Figure 1).

Why should this be so? It seems to us that the areas of greater discrepancy – loosely, where norms might be seen as weaker - reflect both societal change and resource constraint. Many precedents, campaigns, and programs encourage female education, and this is augmented by the observation that, in urban Mumbai at least, girls nowadays usually go to school. In this sense, the descriptive norm favours female education – up to a point – and perhaps the injunctive norm is itself changing. Urban life is expensive and people are time-poor. Women need to be able to manage household expenses and income from their own employment. At the same time, the gendered division between reproductive and productive work has historically meant that domestic parsimony and responsibility are characteristics of a good wife and mother. Whether or not there is increasing permissiveness toward premarital sex, the penalties for young women can be severe. Young men, however, have traditionally been granted the privilege of premarital sexual relationships and, although this licence may have more of a passive than an active character, it is arguably part of their gender role.

There were some limitations to our study. We purposefully interviewed more women than men and we chose an informal settlement which – though very large and occupied by communities of diverse origin – may limit generalisation. The use of vignettes might also have led to a focus on the scenarios that they included, to the exclusion of other possible issues. We have some assurance, however, from the extensive dataset of client records on which the vignettes were based.

Recent reviews have identified community mobilisation as a potentially effective means of reducing violence against women and girls ^43^. There are some models for programs ^44– 50^, all of which aim to change norms, but the evidence for effectiveness is not yet robust ^6^. Several programs have worked to change norms in India. The Samata program seeks to reduce HIV acquisition and child marriage through changing education norms for lower caste girls in Karnataka state. Also in Karnataka, Samvedana Plus addresses violence against sex-working women ^51, 52^. The Yari-dosti program promotes gender equity among young men in low-income communities in Mumbai ^53^, and PRACHAR sought to improve young people’s sexual and reproductive health in Bihar ^54^.

A report on norms around violence against women and girls in rural Bihar ^55^, framed its findings around the ‘real man’ and ‘real woman’ and the ‘good’ or ‘bad’ husband. A real man fulfilled the responsibilities of breadwinner, household head, son, husband, and father. He also made his wife happy, respected her views, did not impose restrictions on her, and – importantly - did not perpetrate violence. A real woman was nurturing, caring for home and children and serving and obeying her husband and his parents. A good husband managed household income responsibly and treated his wife with respect, did not abuse alcohol, and was not violent. A bad husband squandered money, abused alcohol, and perpetrated violence, particularly sexual violence. Perhaps the most interesting aspect of the findings for our work was that intimate partner and domestic violence were generally disapproved of: the injunctive norm was non-violence. Violence was, however, acceptable in some situations – such as infidelity - which might be interpreted differently depending on the perspectives of perpetrator and survivor. Recent evidence suggests that this loophole might be wider if transgression is perceived to be intentional rather than unintentional ^32^.

### Programmatic response 1: exploit the mismatch between injunctive and descriptive norms

Norms are characterized by persistence, punctuated equilibrium and tipping points, compression (the range of individual choices varies less than one might expect), and local conformity within global diversity ^18^. Developing our idea about the utility of dissonance between descriptive and injunctive norms, we think that the idea of building a new norm might be less useful than supporting the emergence of a norm that is already developing. Our hypothesis was that we might exploit the disjunction between descriptive and injunctive norms, focusing program activities on evidence that descriptive norms are changing. In doing so, we might also discuss the relatively weaker sanctions for transgression in this situation. One way to do this might be to identify norms for which disjunction already exists, and then to emphasize the numbers of people who are transgressing: to shift the gaze to a source of information that supports change in descriptive norms ^12^.

There is implicit support for this in the literature: Heise and Manji suggest “communicating change as norms begin to shift.” ^11^ The observation that “norm change is particularly likely in homogenous, tightly knit groups in which there is private dissent against the current norm” ^9^ is also interesting because it seems to imply at least dissonance between injunctive and descriptive norms, if not actual changes in expectations. In a gaming experiment, Bicchieri and Xiao suggested that the driver of conformity with a norm was descriptive: what the individual believed others actually did. Injunctive norms seemed to play a part only when they were in line with this belief ^56^. One possible explanation for this is that punishment for perceived transgression is less likely when many people are transgressing: “Descriptive norms act like magnets, whereas injunctive norms act like bans.” ^9^ From a psychological perspective, emphasizing a descriptive norm that differs substantially from an injunctive norm could be expressed as ‘personalised normative feedback ^57^,’ in which people are shown the discrepancies between their estimates of what is usual and what is actually usual.

### Programmatic response 2: expand the reference group

There seems to be general agreement that interventions should target injunctive norms, which “… make it clear to all members of the community that the particular behavior is not welcome.” ^9^ Perhaps counter-intuitively, a good way to do this may be to use evidence of changes in descriptive norms because “… enough members of the group must believe that enough of its members are adopting the new norm.” ^10^ Our program presents us with an environment in which we can support norm change by providing conduits through which individuals can articulate it. These ‘channel factors’ ^9^ – in our case, community groups, model change agents, and the availability of counselling, family therapy, and legal support – make it easier for individuals to change by providing structure and support ^58^.

We have noticed that discussions of the means to change norms have said little about changing the reference group. Since norms are maintained by sanctions within the reference group ^8^, the idea is to help cement new shared beliefs in the key people from whom the individual takes her cue ^8, 11^. Through no fault of their own, the women we interviewed and, to a lesser degree, the men, looked to small reference groups for guidance. The kinds of interest groups whose activities we facilitate have their own sets of injunctive and descriptive norms and, at least to some degree, either erode the salience of existing reference groups or replace them. A corollary of group behaviour is that an individual can use its symbolic features to either identify herself as a member (in-group) or to identify a group as an entity of which she is decidedly not a member (out-group). Efforts to change norms may therefore look to participants distancing themselves from groups that maintain behaviours seen as unjust, or shifting their identification to groups with more conducive norms ^59^. We would argue that the ubiquity of action groups, communities of interest, and identity politics implies that individuals are able to find safe harbour in a reference group that they have chosen as reflecting their own aspirations and differing from those of others. This idea of expanding the reference group has wide applicability and underlies, for example, participatory programs to improve child survival ^60^, nutrition ^61^, and adolescent sexual and reproductive health in India and the region ^54^, all of which are affected by normative behaviour.

## Conclusions

Our findings have crystallised our thinking about norms in light of 16 years of work to address violence against women and girls in informal settlements in India. We would like to suggest a different way of looking at norm change, on the basis of three propositions. First, as already suggested, we should take advantage of the mismatch between descriptive and injunctive norms, given that descriptive norms intolerant of violence are likely to be magnets for behaviour and that sanctions will become less aggressive as adoption increases.

Second, we should take advantage of the existence of injunctive norms that are already inimical to violence against women and girls. We see this articulated by our interviewees and in population surveys ^62^, but disapproval is already explicit in concentric reference groups that take in the state, the media, and the wider world. The socio-ecologic model tends to be invoked in a negative sense, to emphasise the need for change at multiple levels. It also has positive implications. Societal changes such as new legislation and global influences combine with education, incremental improvements in economic status, and exigencies such as the need to work, to create a milieu favourable to change. What we are seeing across the world – with some exceptions - is gender equity taking on the status of a high-level injunctive norm. This may not be articulated at the level of an individual’s reference group (indeed, this is part of the problem), but it filters down through the strata of the socio-ecologic model and can provide a platform for mobilisation. At the same time, increasing education, mobility, and employment are expected to lead to more friendships outside the family circle, and potentially to exposure to wider cultural changes.

Third, we suggest that the key intervention is not only to make people aware of this, but, in doing so, to induct them into a wider or different reference group. This is central to what community interventions actually do: expand participants’ social world to encompass others whose opinions might differ from those they have been exposed to. This expansion of the reference group takes us into an area of considerable experience and theory that has not yet been reflected substantially in the literature on violence against women and girls: the idea that social capital and, particularly, bridging rather than bonding social capital, or weak rather than strong ties, might be associated with wellbeing. We suggest, therefore, that programs to prevent violence focus on creating new ties for women and girls whose social environments are limited. Further support for this comes from anthropological ideas about mitigating violence through intra-group linkages ^63^. This is already implicit in much of our work, which involves forming and facilitating groups. What has not been explicit is the importance of expanding the reference group to one that has already abandoned harmful injunctive norms and is in the process of challenging them through new descriptive norms.

## Data availability

The data referenced by this article are under copyright with the following copyright statement: Copyright: © 2017 Daruwalla N et al.

Transcripts of focus group discussions and interviews, translated into English, are available at the UK Data Service ReShare ( http://reshare.ukdataservice.ac.uk/), under the title ‘Changing gender norms in the prevention of violence against women and girls in India’, DOI, 10.5255/UKDA-SN-852735. The safeguarded data files are made available to users registered with the UK Data Service under UK Data Archive End User Licence conditions ( https://www.ukdataservice.ac.uk/get-data/how-to-access/registration). The data files are not personal, but – given the subject matter of the interviews and focus groups - the data owner and research ethics committee consider there to be a limited residual risk of disclosure.
