# Supplementary material for: You can’t burn the house down because of one bedbug: a qualitative study of changing gender norms in the prevention of violence against women and girls in an urban informal settlement in India
Source: Wellcome Open Res. 2017 Nov 7;2:48. Originally published 2017 Jul 4. [Version 2] doi: 10.12688/wellcomeopenres.11805.2 (PMC5680532; doi:10.12688/wellcomeopenres.11805.2)
Supplement: Peer review history

# Peer review: Referee response for version 2

**DOI:** 10.21956/wellcomeopenres.14165.r27701

**Published:** 2017-11-09

**Authors:** Beniamino Cislaghi

**Affiliations:** 1 London School of Hygiene & Tropical Medicine, London, UK

This article is a good example of social norms research in the violence field. After the revision, I have no further reservations and have no further comments to make.

I have read this submission. I believe that I have an appropriate level of expertise to confirm that it is of an acceptable scientific standard.

# Peer review: Referee response for version 2

**DOI:** 10.21956/wellcomeopenres.14165.r27702

**Published:** 2017-11-08

**Authors:** Susannah Zietz

**Affiliations:** 1 Department of Health Behavior, University of North Carolina at Chapel Hill, Chapel Hill, NC, USA

I do not have any comments on the revised version.

I have read this submission. I believe that I have an appropriate level of expertise to confirm that it is of an acceptable scientific standard.

# Peer review: Referee response for version 1

**DOI:** 10.21956/wellcomeopenres.12758.r25397

**Published:** 2017-09-11

**Authors:** Beniamino Cislaghi

**Affiliations:** 1 London School of Hygiene & Tropical Medicine, London, UK

This is a powerful article that touches upon the extremely important intersection between intimate partner violence and social norms. Overall this is an interesting article and offers some important insights for practitioners and researchers working at the intersection between social psychology and gender theory. I highlight its three main merits and shortcomings below, and offer a more detailed review in the second part of this comment.

 Three merits of this paper:

  

1. Is located at the intersection between gender theory and social psychology, working on an important conceptual no people’s land that needs to be further explored.
2. Introduces the reader to the important ways in which descriptive and injunctive norms can interact and how their asynchrony can offer opportunities for social change.
3. Conducts an interesting social norms analysis from qualitative data, on the line of previous similar work in this field, that can be used as model for others.

 

 Three shortcomings of this paper:

  

1. It presents the importance of doing social norms work as tightly related to pluralistic ignorance (a phenomenon occurring when most people’s attitudes are opposite to what they believe others’ attitudes to be). But norms and attitudes can be aligned, and yet achieving change requires to address both norms and attitudes.
2. It invites the reader to analyse gendered relations and practices through a social norms lens, but doesn’t integrate a thorough conceptual understanding of the difference between gender norms and social norms. A larger discussion of how gender (roles, norms, schemas, identity) differ would also be helpful.
3. It underplays the role that self-understanding as in- or out-group plays in influencing people’s decision to comply with a norm.

 

**Detailed feedback:**

 This is an important paper and the work is interesting. The work on social norms and violence is still emerging as a field of research and action, and much is left to learn on how the theory can best serve the practice. For this reason, papers such as this one are very welcome.

**Introduction**

 Social norms literature is vast and multifaceted; the authors decided to use the Focus Theory of Normative Conduct developed by Cialdini and colleagues. Their conceptual understanding of social norms could expand to include awareness that other reasons for compliance exist other than normative influence (what the authors call “the influence of a reference group of people important to an individual’s decision-making”, with a phrase that echoes the Theory of Planned Behaviour). The most important of these mechanisms for compliance being: 1) social categorisation, 2) social identity, 3) informational influence (coordination and cooperation); and 4) power ^1^.  The authors could also expand on the strength of the norms they identified. As myself and Heise argued elsewhere ^2^, there is a tendency in the world of global justice and international development to look at social norms as on/off switches, overlooking their varying strength, a point also mentioned in Cislagi & Heise 2016 ^3^ and [report]. The authors make great use of the concept of “pluralistic ignorance”, when norms and individual attitudes are not aligned. This is an insightful concept, but it’s important to stress that the opposite can also (and quite often is) true: social norms and individual attitudes can be aligned; norms can actually shape those attitudes (as scholars working in the gender theory field might argue).

 The authors use gender and social norms almost interchangeably; it would be important to understand if they think they differ at all. The social norms construct they use comes from a social psychology, but much theory around gender norms has been developed by scholars conceptually distant from Cialdini and colleagues.  The authors mention that examples of community mobilisations are rare; they do mention some of these examples and could also mention well-known cases ^4^^-^^7^. In addition, they could look at the case of social norms and intimate partner violence in Southern India discussed in Cislaghi & Bhattacharjee 2017 ^8^.

**Methods**

 Study procedures are very thoroughly explained and well thought. Sometimes, however, the “provisional normative statements” included in Table 1 leave the doubt on whether those statements are all socially normative or morally normative. The difference is sometimes subtle and understandings can overlap.

**Results**

 The results offer a solid qualitative social norms analysis. Authors could have mapped the descriptive and injunctive norms in a table, to show their concordance or discordance. Sometimes it is not completely clear whether authors are looking at norms as defined in the social psychology literature they cite in the intro, or more in the way they have been articulated in applied gender work (for instance at p.8).

**Discussion**

 Some of these findings resonate with a similar study conducted in Southern India and that would be probably be an interesting read for the authors (8). The discussion brings in gender norms as a construct but – as mentioned earlier – more understanding of what gender norms actually are (and how they relate the Cialdini’s social norms used by the authors) would be helpful-

**Conclusions**

 An understanding of the difference between outgroup and ingroup is missing. That is dangerous, because (as the work by Kees Keiser shows) implementing interventions that do not target the right group can increase compliance with the harmful norms with the outgroups (“that message is not for me – I’d better do X even more to make sure people know I am not one of those”). The intervention speaks back to correcting misperceptions, but literature on violence shows that needs to go with local discussions on the nature of harm and protection, because attitudes and norms might be synchronous. As mentioned earlier, the conclusions overstate the importance of pluralistic ignorance: evidence from West Africa suggests that dialogue can increase the mismatch between attitudes and norms or descriptive and injunctive norms required to bring change at scale.

I have read this submission. I believe that I have an appropriate level of expertise to confirm that it is of an acceptable scientific standard, however I have significant reservations, as outlined above.

## References

[1] BellDCCoxML: Social Norms: Do We Love Norms Too Much?. *J Fam Theory Rev*.2015;7(1) : 10.1111/jftr.12059 28-46 10.1111/jftr.12059 25937833PMC4412469

[2] CislaghiB: Four avenues of normative influence. *under review*.10.1037/hea000061829672098

[3] CislaghiB: Measuring Gender-related Social Norms: Report of a Meeting, Baltimore Maryland, June 14-15, 2016. *London: LSHTM*.2017; Reference source

[4] BajajM: Advancing Transformative Human Rights Education. *Brown G, editor. The Universal Declaration of Human Rights in the 21st Century. Cambridge: OPB*.2016; Reference source

[5] CislaghiB: Values Deliberation and Collective Action: Community Empowerment in Rural Senegal. *New York: Palgrave MacMillan*.2016; Reference source

[6] CislaghiB: Expanding the Aspirational Map. *In: Bajaj M, Flowers N, editors. Human rights education : theory, research, praxis. Philadelphia: University of Pennsylvania Press*.2017;ix Reference source

[7] VaitlaB: Social Norms and Girls' Well-Being.2017; Reference source

[8] CislaghiB: Honour and Prestige: The influence of social norms on violence against women and girls in Karnatak, Souht India. *London: STRIVE*.2017; Reference source

# Response

**Published:** 2017-11-09

**Authors:** David Osrin

**Affiliations:** University College London, UK

Thank you for the useful and incisive review. A summary of our responses follows.

 This is a powerful article that touches upon the extremely important intersection between intimate partner violence and social norms. Overall this is an interesting article and offers some important insights for practitioners and researchers working at the intersection between social psychology and gender theory. I highlight its three main merits and shortcomings below, and offer a more detailed review in the second part of this comment.

 Three merits of this paper:

 1. Is located at the intersection between gender theory and social psychology, working on an important conceptual no people’s land that needs to be further explored.

 2. Introduces the reader to the important ways in which descriptive and injunctive norms can interact and how their asynchrony can offer opportunities for social change.

 3. Conducts an interesting social norms analysis from qualitative data, on the line of previous similar work in this field, that can be used as model for others.

 Three shortcomings of this paper:

1. It presents the importance of doing social norms work as tightly related to pluralistic ignorance (a phenomenon occurring when most people’s attitudes are opposite to what they believe others’ attitudes to be). But norms and attitudes can be aligned, and yet achieving change requires to address both norms and attitudes.
2. It invites the reader to analyse gendered relations and practices through a social norms lens, but doesn’t integrate a thorough conceptual understanding of the difference between gender norms and social norms. A larger discussion of how gender (roles, norms, schemas, identity) differ would also be helpful.
3. It underplays the role that self-understanding as in- or out-group plays in influencing people’s decision to comply with a norm.

Detailed feedback:

 This is an important paper and the work is interesting. The work on social norms and violence is still emerging as a field of research and action, and much is left to learn on how the theory can best serve the practice. For this reason, papers such as this one are very welcome.

 Introduction

 Social norms literature is vast and multifaceted; the authors decided to use the Focus Theory of Normative Conduct developed by Cialdini and colleagues. Their conceptual understanding of social norms could expand to include awareness that other reasons for compliance exist other than normative influence (what the authors call “the influence of a reference group of people important to an individual’s decision-making”, with a phrase that echoes the Theory of Planned Behaviour). The most important of these mechanisms for compliance being: 1) social categorisation, 2) social identity, 3) informational influence (coordination and cooperation); and 4) power ^1^.  The authors could also expand on the strength of the norms they identified. As myself and Heise argued elsewhere ^2^, there is a tendency in the world of global justice and international development to look at social norms as on/off switches, overlooking their varying strength, a point also mentioned in Cislagi & Heise 2016and [report]. The authors make great use of the concept of “pluralistic ignorance”, when norms and individual attitudes are not aligned. This is an insightful concept, but it’s important to stress that the opposite can also (and quite often is) true: social norms and individual attitudes can be aligned; norms can actually shape those attitudes (as scholars working in the gender theory field might argue).

*Response*

*Thanks for these excellent criticisms. Responding to them underlies many of the changes we have made to the paper.*

*We have added to the introduction some text on reasons for compliance with norms:*

*“Why we do what we do is complicated and norms are only part of the story. For example, a behaviour might be sustained or prevented by social structures such as laws and institutions, material contributors such as wealth (or lack of it), or the availability of services. It might equally be driven by personal beliefs, self-confidence, and aspirations. From an economic perspective, norms are supported by coordination, social pressure, signalling, and anchoring. Coordination allows individuals to express themselves in shared languages – literally and metaphorically – and benefits both them and the collective. Social pressure encourages individuals not to act purely in their own interest, but that of the collective. Signalling and symbolism allow individuals to identify with (or, equally, indicate their lack of identification with) social groups. Anchoring effectively sets benchmarks for behaviour within a smaller range than what is possible, an example being the ages at which women and men marry.”*

*We have added a mention of the strength of norms we identified in the discussion:*

*“Why should this be so? It seems to us that the areas of greater discrepancy – loosely, where norms might be seen as weaker - reflect both societal change and resource constraint. Many precedents, campaigns, and programs encourage female education, and this is augmented by the observation that, in urban Mumbai at least, girls nowadays usually go to school. In this sense, the descriptive norm favours female education – up to a point – and perhaps the injunctive norm is itself changing. Urban life is expensive and people are time-poor. Women need to be able to manage household expenses and income from their own employment. At the same time, the gendered division between reproductive and productive work has historically meant that domestic parsimony and responsibility are characteristics of a good wife and mother. Whether or not there is increasing permissiveness toward premarital sex, the penalties for young women can be severe. Young men, however, have traditionally been granted the privilege of premarital sexual relationships and, although this licence may have more of a passive than an active character, it is arguably part of their gender role.”*

*We have also tried to make pluralistic ignorance less of a feature of the paper and it is now mentioned on once.*

 The authors use gender and social norms almost interchangeably; it would be important to understand if they think they differ at all. The social norms construct they use comes from a social psychology, but much theory around gender norms has been developed by scholars conceptually distant from Cialdini and colleagues. The authors mention that examples of community mobilisations are rare; they do mention some of these examples and could also mention well-known cases. In addition, they could look at the case of social norms and intimate partner violence in Southern India discussed in Cislaghi & Bhattacharjee 2017.

*Response*

*Thanks again. We have added a clarification on gender norms to the introduction:*

*“The resistance of social norms to change varies. They may be sensitive to changes in social networks and to the influence of individuals who emerge as role models or deviants, and their supporting matrix may be complex. For example, violence against women is unlikely to be sustained by a single norm and often occurs at the intersection of gender norms that are permissive rather than supportive. A gender norm is a kind of social norm that describes shared social expectations of behaviour specific to gender. It tends to emerge from gender ideology and attitudes that have themselves led to socially constructed gender roles; for example, valuing sons over daughters, idealised conceptions of femininity, and traits that signal masculinity. Most of our discussion is about gender norms that legitimise imbalances of power and, by extension, inequalities in access to resources. Glibly, we might think of these as archetypes of the good woman and the real man: constructs that may hinder change rather than actively support violence, but contribute to imbalances of power.”*

*We have included other sources of scholarship in the introduction…*

*“From an economic perspective, norms are supported by coordination, social pressure, signalling, and anchoring. Coordination allows individuals to express themselves in shared languages – literally and metaphorically – and benefits both them and the collective. Social pressure encourages individuals not to act purely in their own interest, but that of the collective. Signalling and symbolism allow individuals to identify with (or, equally, indicate their lack of identification with) social groups. Anchoring effectively sets benchmarks for behaviour within a smaller range than what is possible, an example being the ages at which women and men marry.”*

*… and in the discussion:*

*“Norms are characterized by persistence, punctuated equilibrium and tipping points, compression (the range of individual choices varies less than one might expect), and local conformity within global diversity.”*

*… and:*

*“From a psychological perspective, emphasizing a descriptive norm that differs substantially from an injunctive norm could be expressed as ‘personalised normative feedback’ in which people are shown the discrepancies between their estimates of what is usual and what is actually usual.”*

*We have given examples from India in the discussion:*

*“Several programs have worked to change norms in India. The Samata program seeks to reduce HIV acquisition and child marriage through changing education norms for lower caste girls in Karnataka state. Also in Karnataka, Samvedana Plus addresses violence against sex-working women. The Yari-dosti program promotes gender equity among young men in low-income communities in Mumbai, and PRACHAR sought to improve young people’s sexual and reproductive health in Bihar.”*

 Methods

 Study procedures are very thoroughly explained and well thought. Sometimes, however, the “provisional normative statements” included in Table 1 leave the doubt on whether those statements are all socially normative or morally normative. The difference is sometimes subtle and understandings can overlap.

*Response*

*We have added to the methods section:*

*“While most of the examples were gender norms, a few – “boys will be boys,” “women should not have sex before marriage,” and “it is a good woman’s duty to make her husband’s family happy” - had the character of more general moral norms.”*

 Results

 The results offer a solid qualitative social norms analysis. Authors could have mapped the descriptive and injunctive norms in a table, to show their concordance or discordance. Sometimes it is not completely clear whether authors are looking at norms as defined in the social psychology literature they cite in the intro, or more in the way they have been articulated in applied gender work (for instance at p.8).

*Response*

*Thanks. We made a table, but then thought that a summary diagram might be nicer, and added Figure 1.*

 Discussion

 Some of these findings resonate with a similar study conducted in Southern India and that would probably be an interesting read for the authors (8). The discussion brings in gender norms as a construct but – as mentioned earlier – more understanding of what gender norms actually are (and how they relate the Cialdini’s social norms used by the authors) would be helpful.

*Response*

*We hope that the revisions we have mentioned previously are an adequate response to this comment.*

 Conclusions

 An understanding of the difference between outgroup and ingroup is missing. That is dangerous, because (as the work by Kees Keiser shows) implementing interventions that do not target the right group can increase compliance with the harmful norms with the outgroups (“that message is not for me – I’d better do X even more to make sure people know I am not one of those”). The intervention speaks back to correcting misperceptions, but literature on violence shows that needs to go with local discussions on the nature of harm and protection, because attitudes and norms might be synchronous. As mentioned earlier, the conclusions overstate the importance of pluralistic ignorance: evidence from West Africa suggests that dialogue can increase the mismatch between attitudes and norms or descriptive and injunctive norms required to bring change at scale.

*Response*

*As mentioned before, we have cut down the mention of pluralistic ignorance. We have also added to the discussion in-group and out-group issues, which we found a particularly interesting insight from the reviewer:*

*“A corollary of group behaviour is that an individual can use its symbolic features to either identify herself as a member (in-group) or to identify a group as an entity of which she is decidedly not a member (out-group). Efforts to change norms may therefore look to participants distancing themselves from groups that maintain behaviours seen as unjust, or shifting their identification to groups with more conducive norms. We would argue that the ubiquity of action groups, communities of interest, and identity politics implies that individuals are able to find safe harbour in a reference group that they have chosen as reflecting their own aspirations and differing from those of others.”*

# Peer review: Referee response for version 1

**DOI:** 10.21956/wellcomeopenres.12758.r24577

**Published:** 2017-08-08

**Authors:** Susannah Zietz

**Affiliations:** 1 Department of Health Behavior, University of North Carolina at Chapel Hill, Chapel Hill, NC, USA

The article qualitatively examines social norms around violence against women and girls, particularly descriptive and injunctive norms of violence. The review of this article is organized by article section.

*Introduction*

 The authors provide a comprehensive introduction into the role of social norms, particularly gender norms and the social construction of masculinity, in violence against women and girls. While the authors argue that the evidence for effectiveness of community mobilization interventions is “not yet robust,” I believe it would strengthen the paper to add 1-2 examples of such interventions in urban India. The models for programs that the authors cite are great examples, all but one are from outside India. Though there are numerous challenges to evaluating such interventions, there are a number of community mobilization interventions in India in the literature (to name a few, there have been evaluations from ICRW, Population Council, UNFPA).

 While the study questions are well conceptualized. The hypothesis is actually not a hypothesis for this study, since the study itself is not on an intervention exploiting the disjunction between descriptive and injunctive norms. It is more of a future interest of how the study findings could be applied. I think that a hypothesis is not strictly necessary due to the exploratory qualitative nature of the study. The hypothesis could then be reframed as an interest of the study. The second half of the first paragraph in the second column of page 3 could be moved into the discussion, since it is more on how the results can be applied.

*Methods*

 The authors provide a clear description of the study community. It is clear that the organization has the unique perspective of having strong contextual knowledge of the study population. The authors also have a unique dataset of clients at the crisis and counseling center, generating a comprehensive list of normative statements to inform the qualitative data collection.

 It would be great if there was a small statement on the possible limitations of using vignettes, in that it leads participants to discuss certain scenarios, potentially limiting the topics covered in the discussion. However, we understand that this could be mitigated by using the dataset of clients at the counseling center. It is very much appreciated that you have published your vignette script, it is a great service to other organizations conducting this type of research in India.

 It would also have been great to include a bit more information included on the Framework Analysis approach and how it was applied.

*Results*

 It is not discussed why there more women recruited than men (56 women and only 15 men). It is unclear as to whether this was intentional or not? If intentional, that should be stated. If not, it would be great to have some discussion on the challenges of recruiting men for the study.  

 We would also be careful with comparing Hindu and Muslim women in the second column of page 8 since the sample is limited to 5 women in the in-depth interviews for each.

*Discussion and Conclusion*

 It would be great if the authors could provide some thoughts on why there were more substantial differences between injunctive and descriptive norms around women’s education, control of income and finances, and premarital sexual relationships, but not in the other areas covered in this study. It also seems like the results from the study in Bihar are not integrated into the rest of the discussion. It does not flow with the paragraph before and after it.

 The discussion and conclusion provide a great application of the results to the work that is being done by the organization. It would be great if the authors could also add how these results could also be applied to other work being done in India, perhaps citing a few other types of interventions that could also benefit from the findings of this study.

I have read this submission. I believe that I have an appropriate level of expertise to confirm that it is of an acceptable scientific standard.

# Response

**Published:** 2017-11-09

**Authors:** David Osrin

**Affiliations:** University College London, UK

Thank you for your useful review. A summary of our responses follows.

 The article qualitatively examines social norms around violence against women and girls, particularly descriptive and injunctive norms of violence. The review of this article is organized by article section.

 Introduction

 The authors provide a comprehensive introduction into the role of social norms, particularly gender norms and the social construction of masculinity, in violence against women and girls. While the authors argue that the evidence for effectiveness of community mobilization interventions is “not yet robust,” I believe it would strengthen the paper to add 1-2 examples of such interventions in urban India. The models for programs that the authors cite are great examples, all but one are from outside India. Though there are numerous challenges to evaluating such interventions, there are a number of community mobilization interventions in India in the literature (to name a few, there have been evaluations from ICRW, Population Council, UNFPA).

*Response*

*We found that the discussion section was a good place to add examples of models from India:*

*“Several programs have worked to change norms in India. The Samata program seeks to reduce HIV acquisition and child marriage through changing education norms for lower caste girls in Karnataka state. Also in Karnataka, Samvedana Plus addresses violence against sex-working women. The Yari-dosti program promotes gender equity among young men in low-income communities in Mumbai, and PRACHAR sought to improve young people’s sexual and reproductive health in Bihar.”*

 While the study questions are well conceptualized. The hypothesis is actually not a hypothesis for this study, since the study itself is not on an intervention exploiting the disjunction between descriptive and injunctive norms. It is more of a future interest of how the study findings could be applied. I think that a hypothesis is not strictly necessary due to the exploratory qualitative nature of the study. The hypothesis could then be reframed as an interest of the study. The second half of the first paragraph in the second column of page 3 could be moved into the discussion, since it is more on how the results can be applied.

*Response*

*Thank you for this suggestion. We have moved the hypothesis to the discussion section.*

 Methods

 The authors provide a clear description of the study community. It is clear that the organization has the unique perspective of having strong contextual knowledge of the study population. The authors also have a unique dataset of clients at the crisis and counseling center, generating a comprehensive list of normative statements to inform the qualitative data collection.

 It would be great if there was a small statement on the possible limitations of using vignettes, in that it leads participants to discuss certain scenarios, potentially limiting the topics covered in the discussion. However, we understand that this could be mitigated by using the dataset of clients at the counseling center. It is very much appreciated that you have published your vignette script, it is a great service to other organizations conducting this type of research in India.

*Response*

*We have mentioned this in the discussion in a new paragraph on limitations of the study.*

*“There were some limitations to our study. We purposefully interviewed more women than men and we chose an informal settlement which – though very large and occupied by communities of diverse origin – may limit generalisation. The use of vignettes might also have led to a focus on the scenarios that they included, to the exclusion of other possible issues. We have some assurance, however, from the extensive dataset of client records on which the vignettes were based.”*

 It would also have been great to include a bit more information included on the Framework Analysis approach and how it was applied.

*Response*

*We have added to the paragraph in the methods:*

*“Because focus groups and interviews followed a semi-structured sequence, we began with a list of general coding categories, which we expanded and sub-categorised into a coding tree in a series of team discussions. These categories described types of gender norm, response to transgression, and classification as descriptive or injunctive. We revisited the analysis repeatedly over six months, reviewing individual transcripts and hierarchies of categorical codes in an effort to achieve a higher level of thematic description. We settled on the comparison of descriptive and inductive norms early, but the idea of the importance of reference groups emerged much later.”*

 Results

 It is not discussed why there more women recruited than men (56 women and only 15 men). It is unclear as to whether this was intentional or not? If intentional, that should be stated. If not, it would be great to have some discussion on the challenges of recruiting men for the study.

*Response*

*It was intentional and we have added to the methods paragraph:*

*“A purposive sample of female and male participants represented two age groups (18-30 and 30-55 years) and four localities. We intentionally included more women than men because our preventive program works primarily with women as drivers of norm change.”*

 We would also be careful with comparing Hindu and Muslim women in the second column of page 8 since the sample is limited to 5 women in the in-depth interviews for each.

*Response*

*We agree and have removed mention of this.*

 Discussion and Conclusion

 It would be great if the authors could provide some thoughts on why there were more substantial differences between injunctive and descriptive norms around women’s education, control of income and finances, and premarital sexual relationships, but not in the other areas covered in this study.

*Response*

*We have added to the early part of the discussion:*

*“Why should this be so? It seems to us that the areas of greater discrepancy – loosely, where norms might be seen as weaker - reflect both societal change and resource constraint. Many precedents, campaigns, and programs encourage female education, and this is augmented by the observation that, in urban Mumbai at least, girls nowadays usually go to school. In this sense, the descriptive norm favours female education – up to a point – and perhaps the injunctive norm is itself changing. Urban life is expensive and people are time-poor. Women need to be able to manage household expenses and income from their own employment. At the same time, the gendered division between reproductive and productive work has historically meant that domestic parsimony and responsibility are characteristics of a good wife and mother. Whether or not there is increasing permissiveness toward premarital sex, the penalties for young women can be severe. Young men, however, have traditionally been granted the privilege of premarital sexual relationships and, although this licence may have more of a passive than an active character, it is arguably part of their gender role.”*

 It also seems like the results from the study in Bihar are not integrated into the rest of the discussion. It does not flow with the paragraph before and after it.

*Response*

*We have cut down the discussion of the Bihar study. Its findings remain relevant and we’ve tried to position it best within the narrative.*

 The discussion and conclusion provide a great application of the results to the work that is being done by the organization. It would be great if the authors could also add how these results could also be applied to other work being done in India, perhaps citing a few other types of interventions that could also benefit from the findings of this study.

*Response*

*We have added to the end of the discussion:*

*“This idea of expanding the reference group has wide applicability and underlies, for example, participatory programs to improve child survival, nutrition, and adolescent sexual and reproductive health in India and the region, all of which are affected by normative behaviour.”*
